# Application of Activated Carbon Derived from Seed Shells of *Jatropha curcas* for Decontamination of Zearalenone Mycotoxin

**DOI:** 10.3389/fphar.2017.00760

**Published:** 2017-10-24

**Authors:** Naveen K. Kalagatur, Kumarvel Karthick, Joseph A. Allen, Oriparambil Sivaraman Nirmal Ghosh, Siddaiah Chandranayaka, Vijai K. Gupta, Kadirvelu Krishna, Venkataramana Mudili

**Affiliations:** ^1^Division of Microbiology, Defence Food Research Laboratory, Mysore, India; ^2^Department of Civil Engineering, Bannari Amman Institute of Technology, Sathyamangalam, India; ^3^Center for Life Sciences, Defence Research and Development Organisation – Bharathiar University, Coimbatore, India; ^4^Centre for Nano-Sciences and Technology, Pondicherry University, Puducherry, India; ^5^Department of Biotechnology, University of Mysore, Mysore, India; ^6^Department of Chemistry and Biotechnology, Tallinn University of Technology, Tallinn, Estonia

**Keywords:** activated carbon, mycotoxins, zearalenone, *Jatropha curcas*, decontamination, antidote

## Abstract

In the present study, activated carbon (AC) was derived from seed shells of *Jatropha curcas* and applied to decontaminate the zearalenone (ZEA) mycotoxin. The AC of *J. curcas* (ACJC) was prepared by ZnCl_2_ chemical activation method and its crystalline structure was determined by X-ray diffraction analysis. The crystalline graphitic nature of ACJC was confirmed from the Raman spectroscopy. Scanning electron microscope showed the porous surface morphology of the ACJC surface with high pore density and presence of elemental carbon was identified from the energy dispersive X-ray analysis. From Brunauer–Emmett–Teller (BET) analysis, S_BET_, micropore area, and average pore diameter of ACJC were calculated as 822.78 (m^2^/g), 255.36 (m^2^/g), and 8.5980 (Å), respectively. The adsorption of ZEA by ACJC was accomplished with varying contact time, concentration of ZEA and ACJC, and pH of media. The ACJC has adsorbed the ZEA over a short period of time and adsorption of ZEA was dependent on the dose of ACJC. The effect of different pH on adsorption of ZEA by ACJC was not much effective. Desorption studies confirmed that adsorption of ZEA by ACJC was stable. The adsorption isotherm of ZEA by ACJC was well fitted with Langmuir model rather than Freundlich and concluded the homogeneous process of sorption. The maximum adsorption of ZEA by ACJC was detected as 23.14 μg/mg. Finally, adsorption property of ACJC was utilized to establish ACJC as an antidote against ZEA-induced toxicity under *in vitro* in neuro-2a cells. The percentage of live cells was high in cells treated together with a combination of ZEA and ACJC compared to ZEA treated cells. In a similar way, ΔΨ_M_ was not dropped in cells exposed to combination of ACJC and ZEA compared to ZEA treated cells. Furthermore, cells treated with a combination of ZEA and ACJC exhibited lower level of intracellular reactive oxygen species and caspase-3 compared to ZEA treated cells. These *in vitro* studies concluded that ACJC has successfully protected the cells from ZEA-induced toxicity by lowering the availability of ZEA in media as a result of adsorption of ZEA. The study concluded that ACJC was a potent decontaminating agent for ZEA and could be used as an antidote against ZEA-induced toxicity.

## Introduction

Mycotoxins are poisonous metabolites of fungi that infest agricultural commodities at various stages of pre-harvesting and post-harvesting sessions under a wide range of weather conditions (Iqbal et al., 2016). The most important fungi responsible for contamination of mycotoxins are *Aspergillus, Penicillium, Fusarium, Alternaria*, and *Stachybotrys* (Bräse et al., 2013). The Food and Agriculture Organization (FAO) has assessed that about one fourth of agricultural commodities are affected by fungi and mycotoxins worldwide (Dall’Asta and Berthiller, 2015). Mycotoxins have a deleterious effect on the well-being of humans and livestock and their occurrence pose a great challenge concerning the food safety (Gamliel et al., 2017). Consequently, the European Union nations (EU), FAO, Joint FAO/WHO Expert Committee on Food Additives (JECFA), and many other countries have established certain regulatory limits over mycotoxins at international trade (Van Egmond et al., 2007). Therefore, the occurrence of mycotoxins in agricultural commodities is a foremost worry for farmers, consumers, and government (Udomkun et al., 2017). Till date, approximately 1000 mycotoxins were identified and their number will increase in next few years with the innovation of newer analytical techniques (Bräse et al., 2013). The five major mycotoxins that contaminate quite regular in agricultural products are aflatoxin B1, fumonisin B1, deoxynivalenol (DON), ochratoxin A, and zearalenone (ZEA) (Richard, 2007).

The ZEA is also well known as RAL or F-2 toxin and exclusively known estrogenic mycotoxin. It is heat resistant, color and odorless, and white crystalline solid substance (Zinedine et al., 2007). The ZEA is produced by a large class of secondary metabolite pathway called polyketide synthases (PKSs) by *Fusarium* spp. including *F. cerealis, F. crookwellense, F. culmorum, F. equiseti, F. graminearum* (*Gibberella zeae*), and *F. semitectum*. These are prevalent soil-borne fungi and widely distributed in temperate and warm climates worldwide, and are regular contaminants of cereals (Zinedine et al., 2007). The accessible data across worldwide point out that maize and its by-products are the most prominent with a high occurrence of ZEA. Though, barley, oats, sorghum, rice, rye, soybean and wheat, and its by-products are found occasionally contaminated with ZEA (European Food Safety Authority [EFSA], 2004). Sometimes, ZEA has been detected in the spices, flavored ingredients, Ayurveda and herbal products, fermented products, vegetarian edible oils, beverages, and drinking water (Gromadzka et al., 2008). The food intake by means of meat and other by-products of animals fed with ZEA contaminated feed has a great health concern. The flesh of several species including chicken, turkey, duck, rabbit, pig, and cow was found to carry over low levels of ZEA and its metabolites (Creppy, 2002). The ZEA exhibits relatively low acute toxic effects and its oral LD50 doses are >2000–20,000 μg/kg of body weight in mice, rats, and guinea pigs, and it was more toxic by intraperitoneal administration (Zinedine et al., 2007). The ZEA mainly affects the female reproductive system, causes immature births, abortions, and vulvovaginitis in livestock, and occasionally may be liable for hyperestrogenism in humans too (Vejdovszky et al., 2017). The International Agency for Research on Cancer (IARC) has checked the mutagenicity of ZEA under *in vitro* and recommended as group 3 carcinogen (IARC, 1999). In the recent studies, the toxicity of ZEA was well explored and revealed the hepatotoxicity, neurotoxicity, immunotoxicity, endocrine toxicity, carcinogenicity, and genotoxicity features (Venkataramana et al., 2014; Kowalska et al., 2016). In the present scenario, decontamination strategies to eliminate the ZEA from contaminated agricultural commodities are much needed to maintain food safety, minimize economic loss, and rescue contaminated food products. Wide varieties of decontamination methods including physical, chemical, and biological methods were applied to reduce toxic levels of ZEA (Zinedine et al., 2007; Kalagatur et al., 2015; Gottardi et al., 2016; Kumar et al., 2016). Among the physical decontamination methods, the addition of nutritionally inert sorbents to food and feed matrices is the most recent technique that has been widely suggested to reduce the toxicity of ZEA. Most of the studies connected to the mitigation of ZEA mycotoxicosis by the usage of adsorbents are primarily focused on aluminosilicates comprising of clays. Unfortunately, application of aluminosilicates for the mitigation of ZEA was a minute success. Avantaggiato et al. (2014) reported that activated carbon (AC) and cholestyramine were good adsorption agents of ZEA and can be applied as a feed additive to avoid hyperestrogenism in livestock. However, the high cost of AC and cholestyramine would make their use economically unaffordable (Galvano et al., 2001). Recently, development of functional nanomaterials and AC with novel physicochemical properties using inexpensive, easily available, and environmentally friendly raw materials is highly attracted research area (Kumar and Sharma, 2008; Patra and Baek, 2017). Especially, AC of *Jatropha curcas* has found to have extensive application in decontamination of a variety of toxic environmental contaminants (Hsu et al., 2014).

*Jatropha curcas* is a shrub, belongs to the family Euphorbiaceae, and native of a Central part of America and Mexico. Nowadays, it is a highly cultivated plant in tropical and sub-tropical parts worldwide. *J. curcas* is resistant to saline and drought and can adapt to gravelly, saline, low fertile, and arid desert conditions (Chen et al., 2016; Lama et al., 2016). The seeds of *J. curcas* comprise around 20% saturated and 80% unsaturated fatty acids and yield an average of 34.4% oil that can be used as a biodiesel. The oil is non-edible and widely used in the preparation of candles, cosmetics, detergents, and coloring agents. The oil cake was used in the preparation of synthetic fibers and plastics (Kumar and Sharma, 2008). The seed shell also contains high amounts of cellulose, hemicelluloses, and lignin and can be a rich source of AC (Hsu et al., 2014). In addition, *J. curcas* presented many medicinal benefits, the latex alkaloid is rich in jatrophine and has potent anticancer activity, and latex was found effective in the treatment of skin, paralysis, and rheumatoid ailment. The tender twigs and leaf juice were used as a medication for a toothache and piles, respectively, and root extracts were used as an antidote to poisonous snake bites (Kumar and Sharma, 2008). Recently, phorbol ester fraction of *J. curcas* seed oil has found an innovative role as bio-insecticide in crop protection (Ratnadass and Wink, 2012). Recently, Chauhan et al. (2016) has synthesized silver particles with leaf extract *J. curcas* and suggested to use as an antibacterial agent on a variety of food-borne bacteria. To the best of our knowledge, the decontamination of ZEA using AC derived from *J. curcas* is not reported yet. In this regard for the first time, we report the synthesis of porous AC with the large specific surface area by facile carbonization of seed shell of *J. curcas* and evaluation of its biological activities and effectiveness in ZEA decontamination.

## Materials and Methods

### Chemicals and Reagents

The ZEA, dichloro-dihydro-fluorescein diacetate (DCFH-DA), rhodamine 123, caspase-3 kit, and 0.22 μm of syringe filters were obtained from Sigma–Aldrich (Bengaluru, India). Acetonitrile, methanol, dimethyl sulfoxide (DMSO), and analytical grade water were obtained from Merck (Bengaluru, India). Dulbecco’s modified Eagle’s medium (DMEM), citrate buffer solution (CBS), an antibiotic solution of penicillin and streptomycin, fetal bovine serum (FBS), and Dulbecco’s phosphate buffered saline (D-PBS) were obtained from HiMedia (Mumbai, India). Live/dead cell assay detection kit was obtained from Invitrogen Detection Technologies (Bengaluru, India), and plastic ware for cell culture was obtained from Nunc (Bengaluru, India).

### Synthesis of Activated Carbon

The seeds of *J. curcas* was collected from Tamil Nadu region, India, and washed thoroughly with distilled water, and dried for 2 weeks at room temperature. The husk was separated from the seeds and crushed into a fine powder at a particle size of 500 μm with a micro hammer mill. A quantity of 450 g fine powder was blended with hot distilled water containing 450 g of ZnCl_2_ for 1 h and dried out at 60 ± 5°C in a hot-air oven. The leftover material was filled in a steel container with a tight lid and repacked with another concentric steel container. The linear empty space of the container was filled with sand in the form of layer-by-layer up to outer container brim. The arrangement for carbonization was made under traces oxygen between the voids of material and reaction was executed at 800 ± 5°C for 60 min in a furnace. Following, reaction mixture was cooled and excess ZnCl_2_ was subjected to leaching out by immersing in a hot solution of 1 M HCl at 80 ± 5°C for 24 h in the oven. The material was washed with hot water till the chloride was detached (tested by the silver nitrate method) and dried out at 105 ± 5°C in a hot-air oven and filtered through 250–500 μm size filter. The obtained material was designated as AC of *J. curcas* (ACJC) and stored in a desiccator at room temperature under dry place for a further purpose.

### Physicochemical Characterization of ACJC

The physicochemical characteristics of the prepared ACJC were investigated using various analytical techniques including X-ray diffraction (XRD), Raman spectroscopy, Scanning electron microscope–energy dispersive X-ray (SEM–EDX), and Brunauer–Emmett–Teller (BET). The XRD analysis was carried out to understand the structural characteristics of the ACJC using X’Pert PRO X-ray Diffractometer (PANalytical, Spectris Technologies Pvt. Ltd., India) with Cu Kα radiation source (λ = 1.541 Å) in the 2𝜃 range from 10° to 70° with a scanning rate 3°/min. The average crystalline size of the prepared sample was calculated using Scherrer equation (Patterson, 1939). The Raman spectrum of ACJC was recorded using Renishaw’s in Via Raman microscope (Renishaw India, Bengaluru) with spectrometer attachment. Green laser with 514 nm wavelength set in a low power mode (0.5% of maximum power) was used to excite the sample. The surface micro-morphology and chemical configuration of ACJC were assessed using SEM (Quanta 200, ICON Analytical Equipment Pvt. Ltd., United States) attached EDX detector. The precise surface area of ACJC was determined using BET surface area analyzer (Micromeritics TriStar II, United States).

### Application of ACJC for Adsorption of ZEA

#### Experimental Plan

The adsorption of ZEA (adsorbate) by ACJC (adsorbent) was studied with various parameters (contact time, concentration of ZEA and ACJC, and pH of media) at room temperature (25 ± 2°C). The ZEA stock was prepared in acetonitrile (10 mg/mL) and further test concentration of ZEA was made in buffer solutions (CBS and D-PBS). The CBS was used as media to carry out adsorption studies at pH 3, 4, 5, and 6 while D-PBS was used as media for adsorption at pH of 7, 8, and 9.

#### Adsorption Studies

The impact of contact time (min) on adsorption of ZEA by ACJC was measured with different concentration of ZEA (25, 50, 75, and 100 μg/mL) and 1 mg/mL of ACJC at pH 7 for a period of 3 h. A volume of 50 μL sample solution was collected at an appropriate time interval and used for the quantification of ZEA. The time at which adsorption of ZEA was saturated defined as adsorption equilibrium time and used as a constant time period for further studies. To determine the influence of pH on adsorption of ZEA by ACJC, the experiment was executed at different pH (3, 4, 5, 6, 7, 8, and 9) with 1 mg/mL of ACJC and different concentration of ZEA (25, 50, 75, and 100 μg/mL) at adsorption equilibrium time. The impact of altered concentration of ACJC (0.5, 1, 1.5, 2, 2.5, 3, 3.5, and 4 mg/mL) on adsorption of ZEA (100 μg/mL) was studied at pH 7 and adsorption equilibrium time. In all the experimental setups, the test samples were vortexed at 145 rpm and at room temperature under dark throughout the experiment. At an appropriate time interval, the required test sample was collected and filtered by 0.22 μm syringe filter and the filtrate was used for the quantification of ZEA employing HPLC. The ZEA test solution prepared in buffer solutions without ACJC was referred as a control throughout the experiment.

#### Desorption Studies

The stability of ZEA adsorption by ACJC was determined by changing the pH of media. The ZEA adsorption study was carried out at pH 7 with 25, 50, 75, and 100 μg/mL of ZEA and 1 mg/mL of ACJC for a period of adsorption equilibrium time. Following, the adsorbent was removed from the test sample by centrifugation at 4000 rpm and suspended at pH 3 and vortexed for 30 min at 145 rpm under dark. The supernatant of adsorption and desorption studies were used for the quantification of ZEA.

#### Quantification of ZEA by HPLC

The quantification of ZEA was carried out using HPLC (Shimadzu, Japan) attached with Luna C18 column, 5 μm thickness, and length of 250 mm × 4.6 mm (Phenomenex, United States) as per our previous reported methodology (Kalagatur et al., 2015). The quantity of ZEA adsorbed by ACJC was determined as the difference between the quantity of ZEA in control and supernatant of the test sample with ACJC. The amount of ZEA bound to the ACJC was determined by the following formula:

(1) $$\begin{array}{c} q_{e}=[(C_{0}-C_{e})V]/m \end{array}$$

where *q*_e_ was amount of ZEA adsorbed by specific quantity of ACJC (μg/mg), *C*_0_ was initial amount of ZEA in supernatant of control (μg/mL), *C*_e_ was amount of ZEA left over in supernatant of test sample at equilibrium time (μg/mL), *V* was volume of experimental solution (mL), and *m* was quantity of ACJC (mg).

#### Adsorption Isotherms

The obtained data of adsorption studies were applied to optimize the design of adsorption by equilibrium isotherms of Freundlich and Langmuir models. The Langmuir model was founded on the hypothesis that adsorption was a category of chemical process and the adsorbed layer was measured as unimolecular. The linear form Langmuir model can be represented as follows:

(2) $$\begin{array}{c} \frac{C_{e}}{q_{e}}=\frac{1}{q_{e}b}+\frac{C_{e}}{q_{0}} \end{array}$$

where *q*_0_ signifies the adsorption capacity of ACJC (μg/mg), *q*_e_ was the quantity of ZEA adsorbed by ACJC at equilibrium time (μg/mg), and *b* signifies the energy of adsorption (mL/mg).

The adsorption capacity (*q*_0_) and adsorption energy (*b*) of ACJC were determined from slope and intercept of linear plots of *C*_e_/*q*_e_ versus *C*_e_. The goodness of fit was determined from the correlation coefficient of the fitted curve. The essential characteristics of the Langmuir isotherm can be conveyed by a dimensionless constant called equilibrium parameter (*R*_L_), which signifies the type of isotherm. The *R*_L_ indicates that adsorption to be favorable (0 < *R*_L_ < 1), linear (*R*_L_ = 1), irreversible (*R*_L_ = 0), and unfavorable (*R*_L_ > 1) (El-Sayed et al., 2014). The *R*_L_ was deducted using the following formula:

(3) $$\begin{array}{c} R_{L}=1/(1+bC_{0}) \end{array}$$

The Freundlich isotherm model assumes that ratio of adsorbent adsorbed to the amount of adsorbate is a function of the solution. The empirical model was consistent with the characteristic of heterogeneous surfaces and an exponential distribution of active centers. Thus, the amount of adsorbent adsorbed (*q*_e_) is correlated to the to the equilibrium concentration of adsorbate in solution (*C*_e_). The heterogeneous sorption model of Freundlich can be represented as follows:

(4) $$\begin{array}{c} q_{e}=K_{F}C_{e}^{1/n} \end{array}$$

The K_F_ determines the favorability of the adsorption process. The linearized equation by taking logarithm both sides can be represented as follows:

(5) $$\begin{array}{c} \ln q_{e}=\ln K_{F}+1/n\ln C_{e} \end{array}$$

### *In Vitro* Application of ACJC as an Antidote against ZEA-Induced Toxicity

#### Design of Experiment

In the final aim of the study, adsorption property of ACJC was utilized to establish ACJC as an antidote against ZEA-induced toxicity under *in vitro* studies. The antidote application of ACJC against ZEA-induced toxicity was assessed in neuro-2a cells (neuroblastoma of *Mus musculus*) by determining the live and dead cells, intracellular reactive oxygen species (ROS), mitochondrial membrane potential (ΔΨ_M_), and caspase-3 activity. The neuro-2a cell line was attained from the National Centre for Cell Science (NCCS), Pune, India. The cells were grown in DMEM added with 10% FBS, 50 mU/mL of penicillin, and 50 μg/mL of streptomycin under a moist environment of 5% CO_2_ and 95% air at 37°C. The cells were grown in 75 cm^2^ flasks, media were changed at an alternative days, and confluent cells were used for the experiment. The experiment was designed into four groups: Group I: Cells were exposed only with D-PBS (control). Group II: Cells were exposed with ZEA (25 μg/mL) in D-PBS. Group III: Cells were exposed to 1 mg/mL of ACJC in D-PBS. Group IV: ZEA (25 μg/mL) and ACJC (1 mg/mL) were blended in D-PBS and exposed to the cells.

#### Live/Dead Cell Assay

The live/dead dual staining assay was carried out using calcein AM and ethidium homodimer-1 fluorescent dyes as per instructions of the manufacturer (Haugland et al., 1994). Approximately, 5 × 10^4^ cells were seeded in 12-well plate and the cells were allowed to settle for 6 h. The cells were exposed to ZEA, ACJC, and the combination of ZEA and ACJC as mentioned in the Section “Design of Experiment” and incubated for 6 h. Following, cells were washed with D-PBS for twice and stained with 2 μM of calcein AM and 4 μM of ethidium homodimer-1 prepared in D-PBS for 15 min. Subsequently, cells were washed with D-PBS for twice and images were captured under filters of green fluorescent protein (GFP) and red fluorescent protein (RFP) using an inverted fluorescence microscope (EVOS, Life Technologies, United States). The intensity of fluorescence was recorded at excitation and emission of 485 and 530 nm for calcein AM and 530 and 645 nm for ethidium homodimer-1 using multiplate reader (Synergy H1, BioTek, United States), respectively. The percentages of live and dead cells were calculated using the formula (Garcia-Recio et al., 2015):

$$\begin{array}{c} \mathrm{Live}\;\mathrm{cells}\;(\%)=\frac{F{\left(530\right)}_{\mathrm{sam}}-F{\left(530\right)}_{\min}}{F{\left(530\right)}_{\max}-F{\left(530\right)}_{\min}}\times 100\\ \mathrm{Dead}\;\mathrm{cells}\;(\%)=\frac{F{\left(645\right)}_{\mathrm{sam}}-F{\left(645\right)}_{\min}}{F{\left(645\right)}_{\max}-F{\left(645\right)}_{\min}}\times 100 \end{array}$$

where *F*(530)_sam_ and *F*(645)_sam_ were fluorescence of sample labeled with calcein AM and ethidium homodimer-1 at 530 and 645 nm, respectively. *F*(530)_min_ was fluorescence of sample at 530 nm labeled with ethidium homodimer-1 alone, where all cells were alive. *F*(530)_max_ was fluorescence of sample at 530 nm labeled with calcein AM alone, where all cells were alive. *F*(645)_min_ was fluorescence intensity of the sample at 645 nm labeled with calcein AM alone, where all cells were dead. *F*(645)_max_ was fluorescence intensity of the sample at 645 nm labeled with ethidium homodimer-1 alone, where all cells were dead.

#### Determination of Intracellular ROS

The estimation of intracellular ROS molecules of cells was carried out using DCFH-DA fluorescent probe (Venkataramana et al., 2014). Approximately, 5 × 10^4^ cells were seeded in 12-well cell culture plate and incubated for 6 h to attach the cells to the plate. Following, cells were exposed to different experimental groups as mentioned in the Section “Design of Experiment” and incubated for 6 h. The cells were washed with D-PBS for twice and stained with 5 μM of DCFH-DA for 5 min. Subsequently, cells were washed with D-PBS for twice and fluorescent microscopic images of cells were captured under GFP filter using an inverted microscope (EVOS, Life Technologies, United States) and optical density was measured with the multiplate reader (Synergy H1, BioTek, United States) at excitation and emission of 495 and 550 nm, respectively. The results were expressed in percentage of fluorescence released with respect to the control.

#### Determination of Mitochondrial Membrane Potential (ΔΨ_M_)

The ΔΨ_M_ of the cell was determined by rhodamine 123 fluorescent probe (Venkataramana et al., 2014). Approximately, 5 × 10^4^ cells were seeded in 12-well cell culture plate and allowed to settle for 6 h and treated with differential experimental groups as mentioned in Section “Design of Experiment” and incubated for 6 h. Following, cells were washed for twice with D-PBS and stained with 5 μM of rhodamine 123 for 15 min. Again, cells were subjected to washing for twice with D-PBS and images were captured under GFP filter using an inverted fluorescent microscope (EVOS, Life Technologies, United States) and optical density was monitored at 534 nm using a multiplate reader (Synergy H1, BioTek, United States). The results were expressed in percentage of ΔΨ_M_ with respect to the control.

#### Analysis of Apoptosis by Caspase-3 Kit

The caspase-3 kit was used to estimate the caspase-3 and apoptosis (Riss et al., 2016). Approximately, 5 × 10^4^ cells were seeded in 12-well cell culture plate and allowed to settle for 6 h and treated with differential experimental groups as mentioned in the Section “Design of Experiment” and incubated for 6 h. Following, the cells were washed with D-PBS for twice and treated with the caspase-3 fluorimetric kit as per technical bulletin of the manufacturer and optical density was recorded at an excitation and emission of 360 and 460 nm, respectively. The caspase-3 levels were calculated from a standard of the fluorescent molecule 7-amino-4-methyl coumarin (AMC) release. The results were expressed in percentage of caspase-3 release with respect to the control.

### Statistical Analysis

The experiments were undertaken individually for six times (*n* = 6) and results were expressed in mean ± SD. The statistical analysis was executed by one-way ANOVA following Dunnett’s test and *p*-value < 0.05 was stated as statistically significant (^∗^*p* < 0.05, ^∗∗^*p* < 0.01, and ^∗∗∗^*p* < 0.001).

## Results and Discussion

### Synthesis and Physicochemical Characterization of ACJC

Since last decade, researchers around worldwide have been highly focused on to develop low-cost AC from the abundant and cheaply accessible natural resources. The plant sources are a highly suitable for synthesis of AC due to its high cellulose and hemicellulose content, and the cellulose and hemicellulose have exhibited a high potential sorption competence toward a variety of toxic compounds (Correa et al., 2017; Ooi et al., 2017). Henceforth, in the present study, cellulose- and hemicellulose-rich seed shells of *J. curcas* were used as a raw material to synthesize of AC by ZnCl_2_ chemical activation method.

**FIGURE 1:**
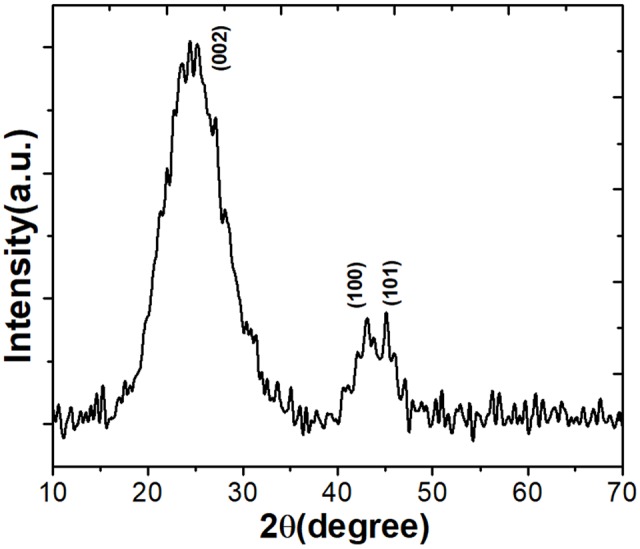
The X-ray diffraction (XRD) pattern of the prepared activated carbon (AC) of *J. curcas* (ACJC).

The crystalline phase of synthesized ACJC was identified from the XRD result (**Figure 1**). The diffraction patterns agree well with the standard JCPDS file (41–1487), which confirmed the formation of crystalline structures of the graphitic carbon. The obtained diffraction peaks are indexed to the corresponding peaks for (002), (100), and (101) crystal planes of graphitic carbon. The presence of a sharp peak around 26° has confirmed the crystalline structure of ACJC which portrays the superior alignment of disordered graphitic carbon layers to form the crystalline turbostratic structure (Li et al., 2007). The average crystallite size of ACJC was calculated from the FWHM value of the highly intense peak corresponding to the (002) crystal plane. It is found that the ACJC has an average crystallite size of 1.07 nm. The peak splitting observed in the X-ray diffraction peaks are due to the transition of carbon from a highly symmetric phase to a lower one. It also shows the presence of microporous wall structure of ACJC. The XRD results confirmed that ACJC has a better crystallographic structure as well-organized aromatic carbon that is more stable than amorphous-like carbon and these results were further evaluated by Raman spectroscopic analysis.

**FIGURE 2:**
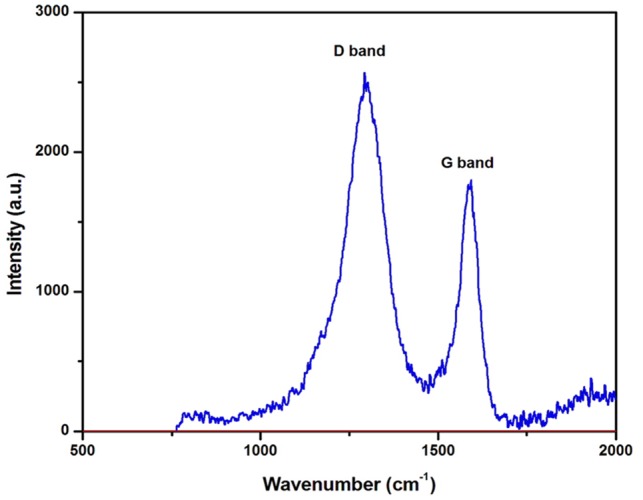
Raman spectroscopy of AC of *J. curcas* (ACJC) was captured at an excitation wavelength of 514 nm using a green laser.

**Figure 2** shows the Raman spectrum recorded by exciting the sample using a green laser with 514 nm excitation wavelength. The Raman spectrum of ACJC elucidates the chemical structure and crystalline nature of the prepared sample. The 514 nm selectively excites the π state of sp^2^ hybridized carbon molecules. The obtained Raman peaks around 1303 and 1589 cm^-1^ are indexed to the D band and G band of the graphitic carbon particles, respectively. The G band represents the peak corresponding to vibrational modes arises due to the stretching of bonds associated with the pairs of sp^2^ hybridized carbon atoms present either in carbon molecular chain or in aromatic ring. The E2g symmetry modes around 1589 cm^-1^ in the G band confirm the presence of highly crystalline and ordered nature of graphitic carbon in ACJC. Compared to the G band of graphite around 1575 cm^-1^, the higher value of peak at 1589 cm^-1^ for the G band of ACJC sample is attributed to the C–C bond bending in the sample due to the porous nature of the ACJC. The D band observed around 1303 cm^-1^ corresponds to the A1g mode called as lattice breathing mode. The presence A1g breathing mode reveals the structural disorder in the sample. Moreover, the peak corresponding to the D3 band absent in the ACJC, which confirms that the amorphous phase of carbon between graphitic layers is absent in the ACJC sample. Hence, it confirms that the ACJC sample is highly crystalline nature (Lazzarini et al., 2016).

**FIGURE 3:**
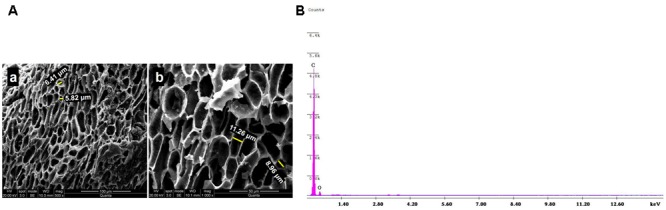
**(A)** SEM images of AC of *J. curcas* (ACJC) were captured at a different magnification of **(a)** 500× and **(b)** 1000×. **(B)** Elemental composition of AC of *J. curcas* (ACJC) was determined by EDX spectrum.

The SEM analysis was undertaken to determine the surface morphology of the synthesized ACJC and micrographs were recorded at different magnifications (500× and 1000×) as shown in **Figure 3A**. The obtained images revealed the honeycomb void structure analogous to the turbostratic morphology of the carbon particles, and this result is in perfect agreement with the structural data obtained from XRD analysis. The ZnCl_2_ chemical activation method has established new microporous as well as widened existing microporous by increasing the volume and surface area of the microporous, and these pores were formed because of ZnCl_2_ evaporation during carbonization. These large volumes of microporous developed by ZnCl_2_ were helpful in engulfing the contaminants. The volume and surface area of microporous depends on the structure of precursor applied for the carbonization purpose.

The elemental analysis of ACJC was carried out using EDX technique and results suggest that the seed shells of *J. curcas* contain a good amount of carbon. The carbonization of seed shells of *J. curcas* by ZnCl_2_ chemical activation has increased the carbon content and indicated successful carbonization of seed shells of *J. curcas* results in the formation of ACJC. **Figure 3B** elucidated the presence of carbon (C) and oxygen (O) in ACJC and wt. % of C and O were 93.36 and 6.64, respectively. A small amount of oxygen is present in the ACJC due to the oxidation of carbon during the heat treatment process.

**FIGURE 4:**
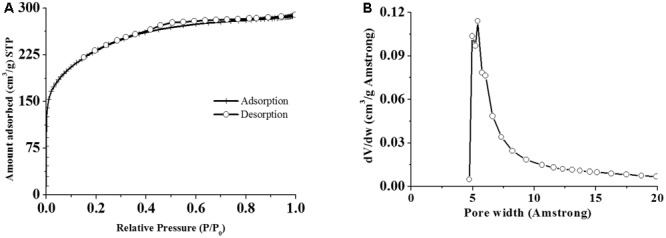
Determination of **(A)** specific surface area and **(B)** pore width of AC of *J. curcas* (ACJC) by BET analysis.

The adsorption capacity of AC depends on volume, size, and specific surface area of the pore (Dizbay-Onat et al., 2017). The specific surface of ACJC was assessed from adsorption experiments on the basis of the BET method, and the micropore volume (*V*) was evaluated by calculating the adsorbate volume. The *t*-curve was analyzed to measure the thickness of the adsorbed film (Senniappan et al., 2016). The BET analysis was helpful in establishing the relationship between surface area and material properties of samples and the effect of activation temperature, activator, impregnation ratio in the formation of pore formation (Ab Ghani et al., 2017). In the present study, ACJC with the greater surface area was obtained by ZnCl_2_ chemical activation at a higher temperature (Liu et al., 2016). The porous structure of ACJC is due to the “swelling effect” caused by the electrolytic action of ZnCl_2_ which breaks the lateral bonds in the cellulose molecules and increases the pore density in the molecular structure of seed shells of *J. curcas*. Therefore, microporosity and surface area of ACJC has increased (Liu et al., 2016). The S_BET_, micropore area, and average pore diameter of ACJC were observed as 822.78 (m^2^/g), 255.36 (m^2^/g), and 8.5980 (Å), respectively (**Figures 4A,B**). In support of our result, IUPAC has also concluded from S_BET_ results that the occurrence of type I isotherm with high pressure at horizontal plateau could result in the development of narrow pore size scattered microporous material. The Fractal dimension, which is a measure of the roughness of a surface of ACJC, was estimated by applying the Frenkel–Halsey–Hill (FHH) equation to N_2_ adsorption isotherms. The results showed that the change in Fractal dimension was insignificant and the complete development of pores on the surface of ACJC was due to the activation of ZnCl_2_ (Carrott and Carrott, 2007).

### Adsorption of ZEA by ACJC

**FIGURE 5:**
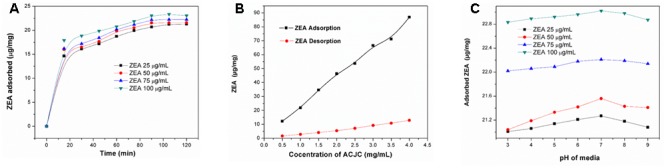
The adsorption of zearalenone (ZEA) by AC of *J. curcas* (ACJC) under various parameters (contact time, concentration of ZEA and ACJC, and pH of media). **(A)** Effect of contact time (min) on adsorption of the different initial concentration of ZEA (25, 50, 75, and 100 μg/mL) by 1 mg/mL of ACJC. **(B)** Adsorption and desorption curve of ZEA for different concentration of ACJC (0.5, 1, 1.5, 2, 2.5, 3, 3.5, and 4 mg/mL) at pH 7 and 3, respectively. The adsorption of ZEA by ACJC was carried out at pH 7 with 100 μg/mL of ZEA for adsorption equilibrium time. Desorption of adsorbed ZEA of different concentration of ACJC was studied at pH 3. **(C)** Effect of different pH (3, 4, 5, 6, 7, 8, and 9) on adsorption of ZEA (100 μg/mL) by 1 mg/mL of ACJC at adsorption equilibrium time of 120 min.

The contact time involved between ACJC (adsorbent) and ZEA (adsorbate) is one of the key factors that considerably influence the adsorption process (El-Sayed et al., 2014). The effect of contact time on the adsorption of ZEA by ACJC was investigated with a different initial concentration of ZEA (25, 50, 75, and 100 μg/mL) and 1 mg/mL of ACJC at pH 7. Based on the kinetic investigations, the plot of ZEA adsorption by ACJC (μg/mg) versus contact time (min) was presented in **Figure 5A**. The results showed the rapid adsorption of ZEA by ACJC for initial 15 min of contact time. Following the contact time, adsorption of ZEA was slow down until equilibrium was obtained and the time taken to obtain adsorption equilibrium time was 120 min. A quantity of 21.27 ± 1.26, 21.56 ± 0.48, 22.21 ± 0.64, and 23.02 ± 0.82 μg of ZEA was adsorbed by 1 mg of ACJC at equilibrium time for the different initial concentration of 25, 50, 75, and 100 μg/mL of ZEA, respectively. The reason behind the adsorption of ZEA by ACJC may be the involvement of strong attraction forces between the positive sites of cationic ZEA and anionic sites of ACJC (Lemke et al., 1998). The study has established ZEA adsorption capability of ACJC over short period of time and it could be effective for decontamination of ZEA.

The effect of different concentration of ACJC (0.5–4 mg/mL) on the adsorption of ZEA (100 μg/mL) was studied at pH 7. The concentration of ZEA adsorption was enhanced with an increase in the dose of ACJC. The kinetic model of ZEA adsorption (μg/mL) versus concentration of ACJC (mg/mL) for a period of adsorption equilibrium time (120 min) was depicted in **Figure 5B**. A quantity of 0.5, 1, 1.5, 2, 2.5, 3, 3.5, and 4 mg/mL of ACJC has adsorbed 12.26 ± 0.20, 23.02 ± 0.82, 34.61 ± 0.52, 46.29 ± 0.95, 53.72 ± 0.67, 66.58 ± 0.86, 71.15 ± 1.46, and 86.92 ± 1.39 μg of ZEA with initial concentration of 100 μg/mL of ZEA, respectively. The quantity of ZEA adsorbed was increased with increasing the dose of ACJC and results showed that it was in the mode of dose-dependent. The reason for the increase in adsorption of ZEA was due to the availability of more number of surface adsorption sites resulting from the increased concentration of ACJC (Sayğılı and Güzel, 2016). The study concluded that the concentration of ACJC was a determinant factor for adsorption of ZEA.

The effect of pH on adsorption of ZEA by ACJC was studied by varying the pH (3, 4, 5, 6, 7, 8, and 9) of media with different concentration of ZEA (25, 50, 75, and 100 μg/mL) and 1 mg/mL of ACJC. The kinetic model of ZEA adsorption by ACJC (μg/mg) versus pH of media at adsorption equilibrium time (120 min) was showed in the **Figure 5C**. The results showed that adsorption of ZEA was noticeably not altered with a change in pH of media. Our result was in accordance with the previous report of Avantaggiato et al. (2014). The pH of media alters the surface charge of adsorbent and the degree of ionization of adsorbate, and it can alter the shift in kinetics and equilibrium of the adsorption process, and it is highly significant during the electrostatic interactions. Generally, the charge of the adsorbate depends on its pK_a_ value and the adsorbate exists mainly in protonated form at pH < pK_a_ and in deprotonated form at pH > pK_a_. Therefore, the effect of pH on adsorption of ZEA by ACJC was dependents on the degree of ionization molecules. However, ZEA has pK_a_ value of 7.6 with diphenolic compound structure and some amount of phenolate anion present in water at pH 7 and the degree of ionization of ZEA was negligible. Therefore, the effect of pH on ZEA adsorption was negligible (Avantaggiato et al., 2014).

Desorption studies of different concentration of ACJC were undertaken with changing the pH of the media. The ZEA adsorption study was carried out at pH 7 with 100 μg/mL of ZEA and different concentration (0.5, 1, 1.5, 2, 2.5, 3, 3.5 and 4 mg/mL) of ACJC for a period of adsorption equilibrium time and desorption studies were executed for period 30 min in the media of pH 3. The amount of ZEA desorbed from adsorbed amount of ZEA was observed as 1.59 ± 0.02, 2.77 ± 0.14, 4.08 ± 0.31, 5.38 ± 0.24, 7.06 ± 0.71, 9.24 ± 0.59, 10.79 ± 0.64, and 12.85 ± 0.72 μg/mL for 0.5, 1, 1.5, 2, 2.5, 3, 3.5 and 4 mg/mL of ACJC, respectively (**Figure 5A**). The results showed that low amount of ZEA was desorbed due to change in the pH of media and suggested that adsorption of ZEA by ACJC was stable with a change in pH of media.

**FIGURE 6:**
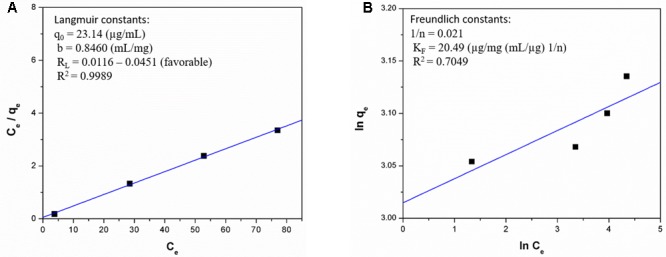
**(A)** Langmuir and **(B)** Freundlich isotherm models for adsorption of zearalenone (ZEA) by AC of *J. curcas* (ACJC).

The adsorption isotherms were deducted by plotting the quantity of ZEA adsorbed per unit of mass of ACJC (*q*_e_) against the quantity of ZEA present in the external phase (*C*_e_) under an equilibrium condition. The obtained data were analyzed by trial version of OriginPro 8.5 software program to fit the isotherm models of Langmuir and Freundlich by the equations (2) and (5), respectively (El-Sayed et al., 2014). The Langmuir model delivered well appropriate correlations for isotherm adsorption plots. The quantity of ZEA adsorbed per unit mass of ACJC was increased gradually by increasing the dose of ACJC. The isotherms presented an exponential graph and a typical Langmuir shape (**Figure 6A**). The isotherm provided a good fit line to experimental adsorption data with small variance and correlation coefficient (*R*^2^) of 0.9989. The separation factor or equilibrium parameter (*R*_L_) of Langmuir isotherm model was obtained from Eq. 3 to evaluate the favorability of ZEA adsorption by ACJC. The *R*_L_ values were observed in between 0.0116 and 0.0451 and it suggested that adsorption of ZEA by ACJC was favorable (El-Sayed et al., 2014). The energy of adsorption (*b*) was observed as a 0.8460 mL/mg and the maximum ZEA adsorption capacity of ACJC was 23.14 μg/mg. The Freundlich isotherm model was constructed with ln *q*_e_ versus ln *C*_e_ following equation (5) (**Figure 6B**). The obtained correlation coefficient of 0.7049 suggested a high degree variance and model was not fitted (El-Sayed et al., 2014). The study concluded that adsorption of ZEA by ACJC was well fitted with Langmuir isotherm model rather than Freundlich, and these results also demonstrated the monolayer sorption of ZEA by homogeneous sites of ACJC (El-Sayed et al., 2014).

The results of the study concluded that ACJC as an efficient decontaminating agent for ZEA. Further investigation should be undertaken to propose a detailed binding mechanism of ZEA with ACJC. Also, regeneration studies should need to carry in detail to recover the adsorbent. So that it will enhance the economic feasibility of the decontamination process. Furthermore, a variety of mycotoxins will be encountered in food and feed matrices, and therefore, the competitive adsorption potential of ACJC should need to be assessed with multi-component mycotoxins.

### *In Vitro* Analysis of ACJC as an Antidote against ZEA-Induced Toxicity

In the therapeutic field, AC has found a diversity of application and used as an antidote to trap the swallowed chemicals, toxins, and overdose drugs in the gut and block their entry into the bloodstream. Therefore, the present study was lastly focused on to evaluate the adsorption feature of ACJC as an antidote against ZEA-induced cytotoxicity under *in vitro* studies. Many studies have proved the cytotoxicity of ZEA and reported that ZEA induces the toxicity by damaging the cellular membrane, generating intracellular ROS molecules, depleting the mitochondrial membrane potential (ΔΨ_M_), and the apoptosis process (Venkataramana et al., 2014). The antidote action of ACJC against ZEA-induced toxicity was checked by assessing the cell membrane damage, generation of ROS molecules, mitochondrial membrane potential, and apoptosis.

**FIGURE 7:**
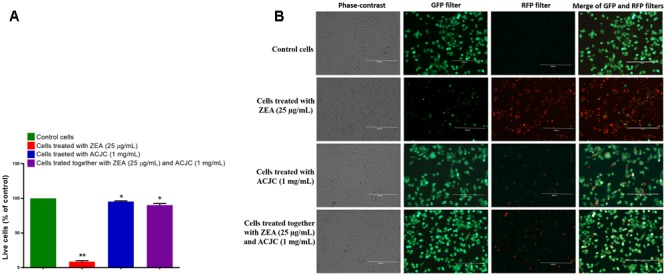
Adsorption property of AC of *J. curcas* (ACJC) was utilized to establish as an antidote against zearalenone (ZEA)-induced toxicity under *in vitro* studies. **(A)** Determination of antidote action of ACJC on ZEA-induced toxicity in neuro-2a cells by live/dead cell assay. (^∗^*p* < 0.05, ^∗∗^*p* < 0.01, and ^∗∗∗^*p* < 0.001). **(B)** Phase-contrast and fluorescent microscopic images of control cells, ZEA exposed cells, ACJC exposed cells, and cells exposed together with ZEA and ACJC at a magnification of 400×.

The live/dead cell assay is a dual staining technique comprising of calcein AM and ethidium homodimer. The calcein AM is cell-permeant dye and in the live cells, calcein AM converted to a green-fluorescent calcein after acetoxymethyl ester hydrolysis by intracellular esterases, and therefore, live cells appear in green under GFP filter, while ethidium homodimer crosses the damaged cell membrane and binds to the nucleus of the cell and emits bright red fluorescence in dead cells under RFP filter (Haugland et al., 1994). In present study, 8.69 ± 3.30% of live cells (green fluorescent cells) were noticed in ZEA treated cells and the percentage of dead cells (red fluorescent cells) were significantly (*p* < 0.05) high in ZEA treated cells compared to control cells and the results evidenced that ZEA-induced the cell death by damaging the cellular membrane (**Figures 7A,B**). On the other hand, cells exposed with ACJC were presented 95.46 ± 2.39% (*p* < 0.05) of live cells and results showed the non-toxicity of ACJC. The cells exposed together with ACJC and ZEA were exhibited 90.28 ± 5.44% (*p* < 0.05) of live cells and results indicated that ACJC has decreased the cytotoxicity of ZEA by lowering the availability of ZEA in media through adsorption.

**FIGURE 8:**
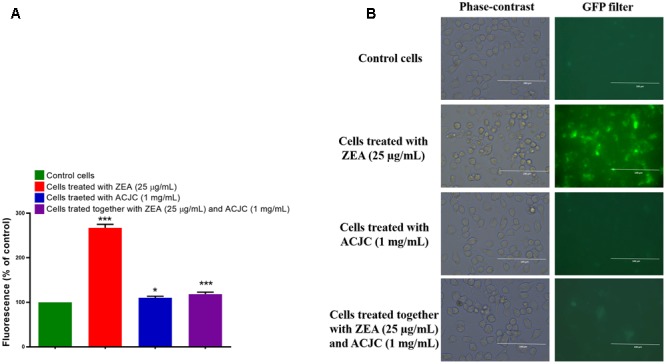
**(A)** Determination of antidote action of AC of *J. curcas* (ACJC) against zearalenone (ZEA)-induced toxicity in neuro-2a cells by estimating the intracellular ROS molecules. (^∗^*p* < 0.05, ^∗∗^*p* < 0.01, and ^∗∗∗^*p* < 0.001). **(B)** Phase-contrast and fluorescent microscopic images of control cells, ZEA treated cells, ACJC treated cells, and cells treated together with ZEA and ACJC at a magnification of 400×.

The antidote action of ACJC on ZEA-induced toxicity in neuro-2a cells was also assessed by determining the intracellular ROS molecules by DCFH-DA probe. The DCFH-DA is a regularly used quantitative method to determine the oxidative species. The DCFH-DA is deacetylated to the non-fluorescent compound by cellular esterases and further oxidize to 2′,7′-dichlorofluorescein (DCF) fluorescent molecules by intracellular ROS (Kumar et al., 2016; Sellamani et al., 2016). In the present study, the percentage of intracellular ROS (267.24 ± 19.46%) was high in ZEA treated cells compared to control (*p* < 0.05). Another hand, percentage of intracellular ROS molecules was not much affected in ACJC treated cells (110.23 ± 8.06) and these results were in line with live/dead cells assay and confirmed the non-toxicity of ACJC (**Figures 8A,B**). The results suggested that ZEA induces the cytotoxicity by oxidative stress through the generation of intracellular ROS molecules (Venkataramana et al., 2014). When cells exposed to a combination of ZEA and ACJC, the percentage of intracellular ROS molecules (118.37 ± 10.79%) was relatively less and not affected (*p* < 0.05). The reason is that ACJC has lowered the availability of ZEA in media by adsorption, and therefore, the level of intracellular ROS molecules was not affected by ZEA.

**FIGURE 9:**
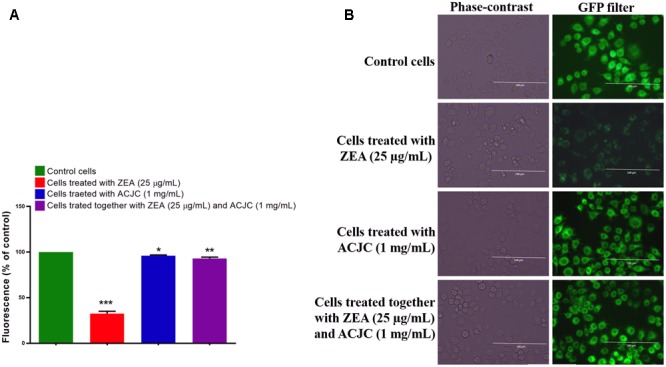
**(A)** Establishment of AC of *J. curcas* (ACJC) as an antidote against zearalenone (ZEA)-induced toxicity in neuro-2a cells by assessing the mitochondrial membrane potential (ΔΨ_M_). (^∗^*p* < 0.05, ^∗∗^*p* < 0.01, and ^∗∗∗^*p* < 0.001). **(B)** Phase-contrast and fluorescent microscopic images of control cells, ZEA treated cells, ACJC treated cells, and cells treated together with ZEA and ACJC at a magnification of 400×.

The antidote action of ACJC on ZEA-induced toxicity in neuro-2a cells was assessed by determining ΔΨ_M_ using probe rhodamine 123. The rhodamine 123 is cell permeable, cationic probe, and emits green fluorescence by the sequestered action of metabolically active mitochondria and measured as an indicator for ΔΨ_M_ of the cell. The depletion in ΔΨ_M_ of cell ceases the synthesis of adenosine triphosphate (ATP) molecules and activates the apoptosis process (Scaduto and Grotyohann, 1999). In the present study, ΔΨ_M_ of cells was significantly (*p* < 0.05) depleted to 32.53 ± 6.17% on exposure of ZEA (**Figures 9A,B**) and another hand, ΔΨ_M_ was noticeably not affected by the treatment of ACJC (96.01 ± 1.85) compared to control (*p* < 0.05). Also, cells treated together with ZEA and ACJC was noticeably (*p* < 0.05) not exhibited depletion in ΔΨ_M_ (92.90 ± 3.66). The results concluded that ACJC has adsorbed the ZEA and lower availability of ZEA in media and therefore, ZEA has failed to deplete the levels of ΔΨ_M_ of the cell.

**FIGURE 10:**
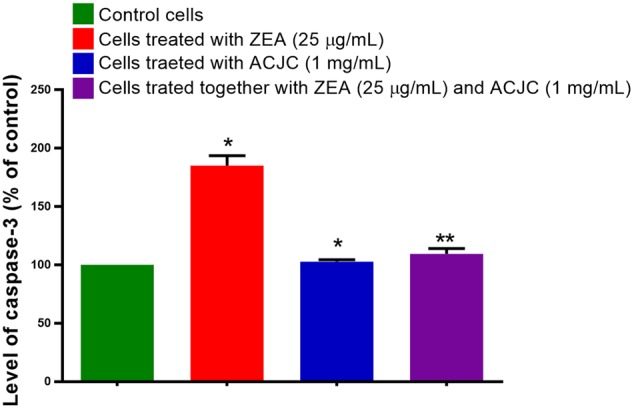
Analyzing the antidote action of AC of *J. curcas* (ACJC) on zearalenone (ZEA)-induced toxicity in neuro-2a cells by determining the caspase-3 levels (^∗^*p* < 0.05, ^∗∗^*p* < 0.01, and ^∗∗∗^*p* < 0.001).

The apoptosis is the common way to induce the death of the cells by a variety of toxic compounds (Venkataramana et al., 2014). In the present study, antidote action of ACJC on ZEA-induced toxicity was determined by measuring the levels of caspase 3 using caspase-3 detection kit following the instructions of the manufacturer. The caspase 3 is an associate of the cysteine-aspartic acid protease family and its successive activation plays a principal role in the accomplishment of cellular apoptosis. The caspase 3 hydrolysis the acetyl Asp-Glu-Val-Asp 7-amido-4-methyl coumarin (Ac-DEVD-AMC) and releases the fluorescent AMC. The concentration of AMC released is directly proportional to the level of caspase 3 and apoptosis (Riss et al., 2016). The concentration of the AMC released was determined from the calibration curve of AMC standards. In the present study, the level of caspase 3 was elevated to 186.53 ± 12.66% on the treatment of ZEA compared to control (*p* < 0.05) and it concluded that ZEA induces the death of cells by apoptosis (**Figure 10**). While cells exposed with ACJC have noticeably (*p* < 0.05) not presented the elevated levels of caspase-3 (102.77 ± 3.31). The cells exposed to a combination of ZEA and ACJC was also noticeably (*p* < 0.05) not exhibited the elevated levels of caspase 3 (109.38 ± 5.93) and this is due to lower availability of ZEA in media by means of adsorption of ZEA by ACJC.

**FIGURE 11:**
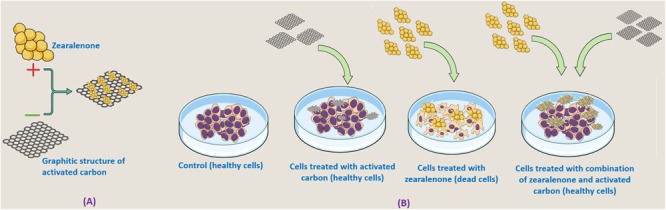
Graphical illustration of **(A)** adsorption of zearalenone (ZEA) by AC of *J. curcas* (ACJC) and **(B)** antidote action of ACJC against ZEA-induced toxicity under *in vitro*.

All the above studies collectively concluded that ACJC has established as an antidote against ZEA-induced cytotoxicity. Furthermore, *in vivo* studies are required to evaluate the application of ACJC as an antidote against ZEA-induced toxicity. However, in support of our study Abdel-Wahhab et al. (2015) has assessed the antidote action of AC on DON-induced cytotoxicity and genotoxicity in rat and concluded that AC could be better antidotes to treat DON-induced toxicity. The JECFA also evaluated the toxicity of AC prepared by the ZnCl_2_ chemical method and recommended as food and feed additive to decontaminate a variety of toxic compounds (JECFA, 2010). Thus, the study concluded ACJC could be used as decontaminating and antidote against the ZEA-induced toxicity and the comprehensive conclusion of the study was presented in graphical design in **Figure 11**.

## Conclusion

We have synthesized the AC from seed shells of *J. curcas* by using ZnCl_2_ chemical activation method. The physicochemical characteristics of the ACJC were characterized by using various analytical technique including XRD, Raman spectroscopy, SEM–EDX, and BET surface analysis. The XRD spectrum indicated that raw seed shells of *J. Curcas* were successfully carbonized into crystalline AC. The formation of the graphitic phase of ACJC was confirmed from the results obtained from Raman spectroscopy analysis. The SEM images depicted that the ACJC has exhibited a large surface area due to its porous structure and morphology and the same has been confirmed from the BET results. Moreover, the presence of carbon and oxygen in the prepared ACJC samples were confirmed by the EDX analysis. The adsorption of ZEA by ACJC was studied with various parameters (contact time, a dose of ZEA and ACJC, and pH of the media). The ACJC has adsorbed the ZEA over a short period of time and adsorption of ZEA was noticed up to the setting up of adsorption equilibrium time. The dose of ACJC has effectively determined the adsorption of ZEA and showed that adsorption was dose-dependent. The different pH of the media was not much influenced by the adsorption of ZEA by ACJC. The desorption of ZEA was studied by changing the pH of the media and little amount of ZEA was desorbed due to change in pH of the media and results suggested that adsorption of ZEA by ACJC was stable. The results of adsorption studies were well fitted with Langmuir adsorption isotherm model and these results evidenced the homogeneous adsorption of ZEA by ACJC. The maximum adsorption of ZEA by ACJC was determined as 23.14 μg/mg. The study concluded that ACJC was efficient for the decontamination of ZEA. The therapeutic application of ACJC against ZEA-induced toxicity was studied under *in vitro* in neuro-2a cells. The results suggested ACJC has successfully protected the cells from ZEA-induced cytotoxicity by lowering the availability of ZEA in media as a result of adsorption of ZEA. The study concluded that ACJC was a potent decontaminating agent for ZEA and could be used as an antidote against ZEA-induced toxicity. Thus, we demonstrated that the economically viable ACJC derived from plant source using an industrially upgradable technology can be a promising substitute for the commercially available AC in the market. We strongly believe that this work will have a great impact on the development of eco-friendly functional AC-based materials from natural resources for decontamination of mycotoxins.

## Author Contributions

NK, KKa, JA, ONG, VM, KKr, SC, and VG designed and interpreted the data of the work. NK, ONG, and VM drafted the work. All authors have approved the final version to be published.

## Conflict of Interest Statement

The authors declare that the research was conducted in the absence of any commercial or financial relationships that could be construed as a potential conflict of interest.
